# Recessive thrombocytopenia likely due to a homozygous pathogenic variant in the *FYB* gene: case report

**DOI:** 10.1186/s12881-014-0135-0

**Published:** 2014-12-17

**Authors:** Hanan Hamamy, Periklis Makrythanasis, Nasir Al-Allawi, Abdulrahman A Muhsin, Stylianos E Antonarakis

**Affiliations:** Department of Genetic Medicine and Development, University of Geneva, Geneva, Switzerland; Department of Pathology, College of Medicine, University of Dohuk, Dohuk, Iraq; Hematology/Oncology unit, JIN Center, Dohuk, Iraq; Service of Genetic Medicine, University Hospitals of Geneva, Geneva, Switzerland

**Keywords:** Platelets, Thrombocytopenia, Familial, Recessive, *FYB* gene, Iraq

## Abstract

**Background:**

Inherited thrombocytopenias (IT) are a heterogeneous group of rare diseases characterized by a reduced number of blood platelets. The frequency of IT is probably underestimated because of diagnostic difficulties and because not all the existing forms have as yet been identified, with some patients remaining without a definitive diagnosis. Exome Sequencing has made possible the identification of almost all variants in the coding regions of protein-coding genes, thereby providing the opportunity to identify the disease causing gene in a number of patients with indefinite diagnoses, specifically in consanguineous families.

**Case presentation:**

Familial thrombocytopenia with small size platelets was present in several members of a highly consanguineous family from Northern Iraq. Genotyping of all affected, their unaffected siblings and parents, followed by exome sequencing revealed a strong candidate loss of function variant in a homozygous state: a frameshift mutation in the *FYB* gene. The protein encoded by this gene is known to be a cytosolic adaptor molecule expressed by T, natural killer (NK), myeloid cells and platelets, and is involved in platelet activation and controls the expression of interleukin-2. Knock-out mice were reported to show isolated thrombocytopenia.

**Conclusion:**

Inherited thrombocytopenias differ in their presentation, associated features, and molecular etiologies. An accurate diagnosis is needed to provide appropriate management as well as counseling for the individuals and their family members. Exome sequencing may become a first diagnostic tool to identify the molecular basis of undiagnosed familial IT. In this report, the clinical evaluation combined with the power and efficiency of genomic analysis defined the *FYB* gene as the possible underlying cause of autosomal recessive thrombocytopenia with small platelet size. This is the first report linking pathogenic variants in *FYB* and thrombocytopenia in humans.

## Background

Inherited thrombocytopenias (IT) are a heterogeneous group of rare diseases characterized by a reduced number of blood platelets. Several genes coding for membrane glycoproteins, cytoskeleton components and intracellular signaling pathways, as well as transcription factors, have been identified as causative in IT, though the pathophysiology remains unknown in some instances [1]. The frequency of IT is probably underestimated because of diagnostic difficulties and because not all the existing forms have as yet been identified, with some patients remaining without a definitive diagnosis [2]. In the last 5 years, nine new genes whose mutations are responsible for thrombocytopenia have been identified. To date, ITs encompass 20 forms caused by mutations of 21 genes with only four syndromes reported to have an autosomal recessive mode of inheritance [3].

High Throughput Sequencing (HTS) has made possible the identification of almost all variants in the coding regions of protein-coding genes, thereby providing the opportunity to identify the disease causing gene in a number of patients with indefinite diagnoses, specifically in consanguineous families [4]. Exome sequencing may become a first diagnostic tool to identify the molecular basis of rare IT [5] as exemplified by the report defining mutations in GFI1B in the causation of macrothrombocytopenia [6].

In this report, we describe a novel autosomal recessive bleeding disorder likely caused by a mutation in the *FYB* gene. FYN binding protein (FYB-120/130), also known as FYB, ADAP (Adhesion- and Degranulation-promoting Adapter Protein), and SLAP-130 (SLP-76-associated phosphoprotein of 130 kDa) and encoded by the *FYB* gene is a cytosolic adaptor molecule expressed by T , natural killer (NK) and myeloid cells and by platelets [7]. This gene is recognized to affect murine T cells and platelet function but has not yet been linked to a human disease. The autosomal recessive bleeding phenotype seen in several members of this highly consanguineous family included petechial rash, mild epistaxis and thrombocytopenia with some decrease in platelet volume. These clinical findings, together with the results of exome sequencing pointed to only one strong candidate gene, the *FYB* gene, known to affect platelet function and causes mild thrombocytopenia in knockout mice [8].

## Case presentation

### Clinical data

**Figure 1:**
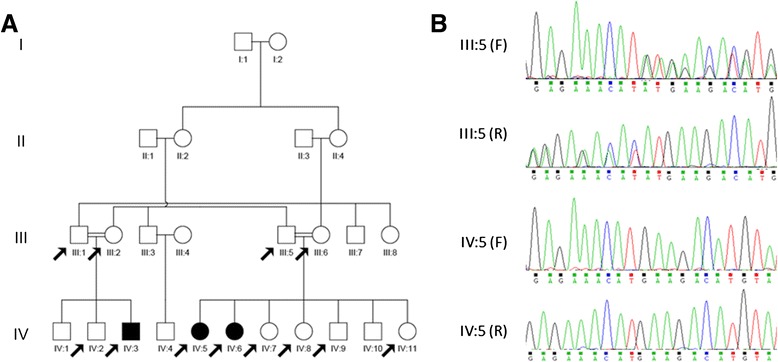
**Pedigree (A) and Sanger sequencing (B). A**: Family’s pedigree. DNA samples were available from individuals marked with arrows. **B:** The segregation of the variant was confirmed in the twelve family members. The figure shows the forward and reverse strand chromatogram of the proband (IV: 5) and her father (III: 5) in the forward (F) and reverse (R) orientation. In the III: 5 s sequences the frameshift is evident in both orientations. The letters underneath are showing the reference sequence. In IV: 5 s sequences no frameshift is observed as expected since the variation is in homozygosity.

Figure 1A shows the pedigree of the highly consanguineous family originating from Northern Iraq. The first proband of the family (IV: 5, Figure 1A) presented in 2003 at the age of 1.5 years with isolated generalized petechial rashes. Investigations revealed normal Hb concentration (128 g/L) and leucocyte count (11.0 × 10^9^/L), with normal differential count and morphology. Platelet count was low at 40 × 10^9^/L and the platelets were of small size with mean platelets volume (MPV) of 6.0 fL [Normal range is 7.0-11.0 fL]. No giant platelets were identified. Bone marrow (BM) aspiration revealed a normocellular marrow, with normal number of megakaryocytes and no abnormal cells. The patient was treated with steroid therapy for various periods and on different occasions for the following 5 years but without any remarkable response (platelets varied between 20–45 ×10^9^/L throughout this period). On re-evaluation in 2008 the patient, showed no gross physical anomalies, no hepatosplenomegaly and no lymphadenopathy. Her physical development was normal, with no skin lesions. Hematologic investigations were similar (Hb: 127 g/L and WBC: 10 × 10^9^/L with normal morphology, platelet count of 25 × 10^9^/L) and again low MPV (5.8 fL). Aspiration showed active BM with normal number of megakaryocytes. The patient was treated with azathioprine with partial response for about 2 months followed by no response and so the drug was discontinued.

In 2009, her younger sister (IV: 6, Figure 1A), 4 years old at that time, developed a similar condition of isolated petechial rash with mild epistaxis. Abdominal ultrasound did not show any abnormalities or hepatosplenomegaly. Blood counts showed a Hb of 126 g/L and WBC of 11.3 × 10^9^/L (with normal differential), platelet count was 32 × 10^9^/L, no giant platelets were seen and MPV was 5.4 fL. Bone Marrow aspiration showed normal number of megakaryocytes.

In the same year, one of their cousins (IV: 3, Figure 1A), a 5 year old male, developed a similar clinical picture of petechial rash as IV:5 and IV:6 with mild mucosal haemorrhage. Blood counts showed a Hb of 127 g/L, WBC 8.2 × 109/L (normal differential and morphology), and platelets count of 34 × 10^9^/L with an MPV of 6.2 fL. BM aspiration showed a normocellular marrow with normal number of megakaryocytes.

None of the patients had immune defects, unusual infections or growth abnormalities and the hematologic indices in the parents of the three patients showed no abnormalities

Since all three affected members in this family were born to consanguineous parents and showed isolated thrombocytopenia with no giant platelets, the most probable diagnosis was familial or autosomal recessive thrombocytopenia. The family provided written informed consent for the genetic analyses performed.

### Investigations

Full blood counts were performed using a hematology analyzer (Beckman Coulter, USA), calibrated daily by quality assurance material provided by the manufacturers. Films for peripheral blood and marrow were stained by Leishman stain for morphology. DNA was extracted from EDTA anticoagulated blood by phenol chloroform method from affected and unaffected family members (marked with arrows in Figure 1).

Genotyping and exome sequencing were performed in the Genetic Medicine laboratory at Geneva University. DNA samples were genotyped using the HumanOmniExpress Bead Chip by Illumina Inc® (San Diego, CA, USA). After filtering for quality, the results were used in order to define the Runs of Homozygosity (ROH) using PLINK [9]. We defined as ROH any region of the genome with 50 consecutive SNPs irrespective of the genomic size, allowing for one mismatch.

The exome of one affected individual (IV: 5, Figure 1A) was captured using the SureSelect Human All Exons v3 reagents (Agilent Inc®). Sequencing was performed in an Illumina HiSeq 2000 instrument. The exome library was indexed, separated into two equal halves and sequenced in two different lanesand the raw results analyzed as described in Makrythanasis et al., [4]. The ROH coordinates, the genotypes and the exome results were processed using an inhouse algorithm (CATCH v1.1, unpublished). CATCH takes additionally into account the family information and assigns every variant to a different class according to how well it respects the segregation of the ROH. The filters used were homozygous exonic and splicing variants (±6 bp from the intron–exon junction) with a minimum allele frequency less than 0.02 in public (dbSNP, 1000 genomes, ESP) and the local database. Only variants found inside ROH and respecting the segregation were kept for further analysis [4].

ROH calculations from the parents revealed in average 70.25 ROH with average size 1.5Mbp and total average length per individual 106Mbp, while in the children the equivalent average values were 80.125 ROH with average size 3.6Mbp and total average length per individual 297Mbp. The increase of the total size of the ROH between the parents and the children is as expected in cases of consanguineous families.

After exome sequencing 91.32% of the RefSeq was covered at least 8x, identifying 19,272 exonic variants of high quality, 9,786 of which were synonymous (50.78%). 8,713 (45.21%) were missense, 56 (0.29%) were nonsense and 326 were indels (1.69%). These numbers are in accordance to what is expected for the attained depth and coverage and the exome capture kit that was used.

**Figure 2:**
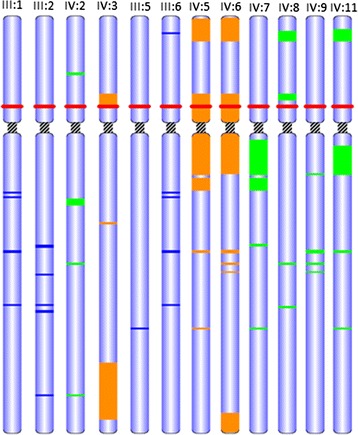
**ROHs identified in chromosome 5 that contains FYB.** The different individuals are shown on top. Parents are noted with blue, affected siblings with orange and unaffected siblings with green. The position of FYB is noted in red. The identified common ROH spanned positions 33622158 to 39735991. In total 20 variants have been found inside this area. 19 of them were excluded due to high reported minimum allele frequency ranging from 0.14 to 0.98. The only novel variant was the one found in FYB.

Only one variant was reported by CATCH respecting the selection criteria and the family segregation (Figure 2), a 2 bp deletion which introduces a stop codon in the *FYB* gene (NM_001465.3:c.1385_1386del: p.(Tyr462*)). Sanger sequencing (Figure 1B) has confirmed the variant in IV:5 and have shown that the other two affected members (IV:3 and IV:6) were also homozygous. As expected the 4 parents were heterozygous for the variant and all the non-affected siblings for whom we had samples (marked as arrows in Figure 1A) were also heterozygous for the variant. Figure 2 focuses on the ROHs identified in chromosome 5 that contains FYB. The different individuals are shown on top. Parents are noted with blue, affected siblings with orange and unaffected siblings with green. The position of FYB is noted in red. The identified common ROH spanned positions 33622158 to 39735991. In total 20 variants have been found inside this area. 19 of them were excluded due to high reported minimum allele frequency ranging from 0.14 to 0.98. The only novel variant was the one found in FYB.

The study was approved by the Bioethics Committee of the University Hospitals of Geneva (Protocol number: CER 11–036).

Inherited thrombocytopenia could be classified according to platelet size, into those with small (MPV < 7 fL), normal (MPV = 7–11 fL) or large/giant (MPV >11 fL) platelets [10]. A number of syndromes of inherited thrombocytopenia with normal and small sized platelets have been described, among which only two syndromes with autosomal recessive mode of inheritance are known. These include congenital amegakaryocytic thrombocytopenia (CAMT, OMIM 604498), and thrombocytopenia with absent radii (TAR, OMIM 274000).

CAMT is characterized by very severe thrombocytopenia giving rise to the risk of life-threatening hemorrhages. The disease progresses to bone marrow aplasia within the first years of life resulting in death if patients do not receive hematopoietic stem cell transplantation [1]. CAMT was excluded as the diagnosis in this family because of the presence of normal number of megakaryocytes in BM in all affected and no pathogenic variant was identified in this gene by exome sequencing. Thrombocytopenia with absent radii (TAR) belongs to the group of inherited hypomegakaryocytic thrombocytopenias associated with skeletal defects [11]. In our family TAR syndrome was excluded because there were no reductions in marrow megakaryoytes and there were no absent radii in any of the patients.

The two syndromes with thrombocytopenia and low MPV, Wiskott-Aldrich syndrome (OMIM 301000) and X-linked thrombocytopenia (OMIM 313900) were not considered in the differential diagnosis for this family because of absence of eczema and repeated infection and because both sexes are affected pointing more to an autosomal recessive mode of inheritance. Most other inherited thrombocytopenias have an autosomal dominant mode of inheritance and high MPV and so were excluded from the differential diagnosis in this family.

The results of exome sequencing identified only one candidate gene (*FYB*, MIM 602731), where homozygous pathogenic variants were present in all affected but none of the non-affected members tested. Parents of affected and all unaffected siblings (marked in arrows in Figure 1A) were heterozygous for the variant. Hematologic investigations of all carriers showed normal results. No human disorder is currently linked to *FYB*.

Structurally, human and murine *FYB* are conserved, with some divergence found mostly near the N-terminal part of the molecule, a region with no apparent homology to other proteins. Conservation is most apparent in the potential functional motifs such as the SH3-like domain and the putative nuclear localization motifs, suggesting that *FYB* may localize to both the cytoplasm and nucleus.

Fyb knockout mice (−/−) are viable, fertile, and show normal growth. Hematopoietic cellularity is normal with the exception of modest significant thrombocytopenia, a 50% reduction in splenic T cells, and mildly decreased thymocyte number [8]. They also, have enlarged spleens as compared with wild-type animals. This may reflect increased removal of platelets from the circulation [12]. Among Fyb−/− mice, 61.8% show rebleeding from tail wounds within 1 minute of initial arrest in comparison to 21.9% of Fyb+/+ mice. Plasma from Fyb−/− mice showed normal thrombin and activated partial thromoboplastin times which may suggest that the higher rebleeding rate is due to platelet dysfunction [13]. Fyb has a role in α_IIb_β_3_ mediated platelet mechanotransduction that promotes F-actin assembly and enables platelet spreading and thrombus stabilization under fluid shear stress [14]. The Fyb knockout mice (−/−) showed thrombocytopenia and normal growth as did the patients with the FYB pathogenic variant, but also showed splenomegaly which was not detected in the human phenotype In light of this evidence we consider *FYB* as the candidate for the thrombocytopenia that was observed in our patients.

## Conclusion

In this report, the clinical evaluation combined with the power and efficiency of genomic analysis defined the *FYB* gene as the possible underlying cause of autosomal recessive thrombocytopenia with small platelet size. The role of the *FYB* gene in causing thrombocytopenia in human will be established when other families with undiagnosed inherited thrombocytopenia show pathogenic variants in this gene. Further studies are needed to elucidate the molecular pathophysiology of this disease and additional families are needed for the full description of the phenotypic manifestations.

## Consent

We confirm that the patient/s (families) have given their consent for the publication of this case report and any accompanying images.
